# Psychometric validation and predictive efficacy of a comprehensive depression risk model for undergraduates

**DOI:** 10.3389/fpsyt.2026.1877501

**Published:** 2026-09-03

**Authors:** Xue Liang, Liuying Lu, Qian Liao, Jinghua Long, Bing Wei, Liying Mo, Yiqian Qin, Caixia Lv

**Affiliations:** 1 The First Affiliated Hospital of Guangxi Medical University, Nanning, China; 2 Department of Epidemiology and Biostatistics, School of Public Health, Guangxi Medical University, Nanning, China

**Keywords:** depressive symptoms, machine learning, predictive model, psychometric validation, sleep quality, social connection, somatic symptoms, undergraduates

## Abstract

**Background:**

Undergraduate depression is prevalent, yet traditional screening is unidimensional and inefficient. We developed a biopsychosocial risk classification model for the cross-sectional identification of current depressive symptoms.

**Methods:**

A cross-sectional study enrolled 898 undergraduates from a medical university in Western China (March–June 2024). The participants were randomized to training (*n* = 719, 80%) and test (*n* = 179, 20%) sets. Assessments included demographics, somatic symptoms (Somatic Symptom Scale), insomnia severity (Insomnia Severity Index, ISI), and depressive symptoms (Patient Health Questionnaire-9, PHQ-9). To avoid circularity, all PHQ-9 items were excluded from predictors; the total score defined the outcome (≥5). Independent risk factors were identified via univariate and multivariate logistic regression. Three models—random forest, XGBoost, and logistic regression—were developed. Performance was evaluated using discriminative metrics (AUC, accuracy, sensitivity, specificity, PPV, NPV, and F1 score), calibration plots, and decision curve analysis. Internal validation utilized fivefold cross-validation and bootstrap resampling (1,000 iterations). Subgroup analyses stratified the results by gender, grade, and somatic symptom severity.

**Results:**

The point prevalence of depressive symptoms (PHQ-9 ≥5) was 45.21% (406/898), which was significantly higher in women (OR = 2.98). Multivariate analysis identified severe somatic symptoms (OR = 37.94), moderate somatic symptoms, and social isolation as key independent risk factors. Excluding PHQ-9 items to avoid circularity, the random forest model achieved an AUC of 0.872 (95% CI: 0.841–0.903), outperforming scale-only (ΔAUC = 0.110, *p* < 0.001) and linear models (ΔAUC = 0.031, *p* = 0.042). Feature importance consistently highlighted somatic distress, insomnia severity, and lack of close friends over emotional items. Calibration was excellent (Hosmer–Lemeshow *p* > 0.05), and decision curve analysis supported net clinical benefit (thresholds 0.2–0.9).

**Conclusion:**

A comprehensive model combining physiological, psychological, and social factors yields excellent cross-sectional discriminative capability and stability for identifying undergraduates currently at risk for depressive symptoms. The proposed three-step clinical pathway (universal screening, targeted re-evaluation, and precision intervention) can facilitate large-scale, early identification in university settings. Due to the cross-sectional design, the term “prediction” is not used in a temporal or causal sense; rather, the model estimates the probability of concurrent depressive symptoms.

## 1 Introduction

### 1.1 Background

Depression has become a significant public health problem affecting the mental health of undergraduates worldwide, with profound impacts on academic achievement, social functioning, and long-term quality of life (1). Epidemiological research indicates that the prevalence of depressive symptoms among Chinese college students ranges between 30% and 50%, a figure exacerbated by the COVID-19 pandemic (2). The pandemic disrupted standard educational models, limited social communication, and created substantial mental pressure, particularly for students who matriculated or graduated during 2020–2021 (3). Undergraduates are in a critical developmental period characterized by identity formation, social adaptation, and academic pressure, during which depression can impair academic performance, foster social isolation, and increase the risk of self-harm and suicidal behaviors (4).

Traditional clinical screening has relied on unidimensional self-report measures (e.g., PHQ-9) and clinical interviews (5). However, these methods have limitations. Unidimensional scales typically focus on emotional symptoms, ignoring the synergistic effects of somatic, psychological, and social factors (6)—for instance, many depressed college students exhibit physical symptoms (e.g., persistent pain, indigestion) before emotional problems, leading to frequent misdiagnosis (7). Clinical interviews, while accurate, are costly and difficult to implement in large-scale student screenings (8). Furthermore, existing tools are often insensitive to cohort-specific stressors, such as pandemic-related disruptions, and individual differences in symptom presentation (9).

Advances in machine learning (ML) offer new paradigms for mental health screening by integrating diverse data types to create efficient and robust predictive models (10). ML algorithms can capture non-linear relationships and complex interactions among variables, overcoming the limitations of traditional statistical methods (11). However, prior ML studies on undergraduate depression share three common weaknesses: (1) insufficient integration of physiological factors (e.g., somatic symptoms, sleep quality), (2) lack of rigorous psychometric validation (e.g., cross-validation, subgroup analysis) to ensure stability and generalizability, and (3) failure to account for cohort differences, such as the variable pandemic impact on different grades (12).

Somatic symptoms and sleep quality are particularly crucial yet often overlooked predictors. Research indicates that physical ailments like fatigue and back pain are not merely correlates but independent risk factors for depression, creating a vicious cycle (13). Sleep problems serve as both markers and prodromal indicators of depression (13). Critically, social connections—a primary protective factor for mental health—were severely disrupted by pandemic-related isolation and must be incorporated into predictive models (14).

Recent computational studies have also contributed to adolescent mental health detection—for instance, the Yuva e-health model provided a digital intervention framework for psychological issues among adolescents (15). Fuzzy logic approaches have been applied to analyze adolescent psychological characteristics (16, 17). An efficient deep learning model using the Harris–Hawk optimizer demonstrated potential in prognostication of mental health disorders (18). Additionally, the SAE-O-BiLSTM model was evaluated for predicting suicidal behavior (19). These studies support the feasibility of machine-learning-based mental health screening, yet they often focus on single techniques or limited indicators, highlighting the need for a comprehensive biopsychosocial model as proposed in our study.

### 1.2 Research gaps

Despite growing interest in ML-based depression prediction, studies on undergraduates lack a comprehensive framework. No studies have simultaneously integrated physiological, psychological, social, and cohort-specific factors. Most models focus on a single domain (e.g., psychological symptoms) (20) and fail to validate performance across key subgroups (e.g., gender, grade, and somatic severity). Furthermore, few studies systematically compare multiple ML algorithms to guide model selection for specific applications (large-scale screening vs. clinical evaluation) (21), and a lack of rigorous psychometric validation (reliability, validity, stability) impedes translation from research to practice (22).

### 1.3 Research objectives

This study aimed to perform the following:

Construct a comprehensive depressive symptoms risk prediction model for undergraduates integrating demographic, somatic, sleep, social, and psychological factors.

Evaluate the psychometric properties (validity, reliability, and stability) of three ML models (random forest, XGBoost, and logistic regression) using rigorous methods.

Identify key risk factors and analyze their mechanisms to inform targeted interventions.

Provide evidence-based practical recommendations for university depression screening tools.

### 1.4 Significance of the study

This study contributes to the literature in three ways. First, it integrates multi-faceted factors (physiological, psychological, and social) for a more complete predictive model, improving precision and clinical relevance. Second, it systematically compares three ML algorithms and validates their performance across subgroups, providing evidence-based guidance for model selection. Third, it employs cohort-specific data (e.g., pandemic effects on different grades) to enhance model applicability to contemporary undergraduates. The results have practical importance for universities to quickly identify high-risk students, design effective intervention programs, and promote undergraduate mental health.

It is important to note that, because this study uses cross-sectional data, the term “prediction” is used throughout only in a statistical classification sense (i.e., estimating the probability of current depressive symptoms from observed features). No causal or temporal predictive claims are made. Any future prospective applications would require longitudinal validation.

## 2 Materials and methods

### 2.1 Study design and participants

A cross-sectional study was conducted with undergraduates at a comprehensive medical university in Western China from March 2024 to June 2024. The participants were selected using stratified sampling by grade (2019, 2020, 2021, and 2023) and major (e.g., clinical medicine, anesthesiology, rehabilitation therapy, medical laboratory technology, pediatrics, forensic medicine, and imaging medicine).

#### 2.1.1 Inclusion and exclusion criteria

Inclusion criteria: (1) Full-time undergraduate student, (2) voluntary participation with written informed consent, and (3) complete data on all questionnaires (missing rate < 10%).

Exclusion criteria: (1) Serious mental disorders (e.g., schizophrenia and bipolar disorder) diagnosed by a psychiatrist, (2) severe physical illness (e.g., cancer and heart disease) affecting mental state, and (3) current psychotropic medication use or participation in other mental health intervention research.

#### 2.1.2 Sample size calculation

Sample size was determined based on the 10:1 events-per-variable rule for ML model development (23). With 20 potential predictors (demographic, somatic, sleep, social, and psychological), a minimum of 200 participants was required. To allow for subgroup analyses and cross-validation, the final sample was 898, with a 4:1 training-to-test ratio (24).

#### 2.1.3 Data partitioning

The participants were randomly assigned to a training set (*n* = 719, 80%) and a test set (*n* = 179, 20%) using stratified random sampling based on gender, grade, and depressive symptoms status to ensure distributional equivalence and reduce selection bias (25).

### 2.2 Data collection tools

#### 2.2.1 Demographic characteristics questionnaire

A self-developed questionnaire assessed the following: gender (male/female), grade (2019/2020/2021/2023), major, height (cm), weight (kg; BMI calculated as weight / (height in meters)^2^), family history of mental illness (yes/no), and number of close friends (0, 1 to 2, 3–5, and ≥6). Close friends were defined as friends with whom one can share personal feelings and seek help.

#### 2.2.2 Somatic symptom scale

This 20-item self-report scale assesses the frequency of somatic symptoms (e.g., stomach pain, back pain, chest pain, dizziness, fatigue, constipation, diarrhea, and shortness of breath) over the past month (26). Each item is rated on a three-point scale (0 = never, 1 = sometimes, and 2 = often). Total scores range from 0 to 40. Somatic problem severity was categorized as follows: none (score = 0), mild (1–10), moderate (11–20), and severe (>20) (26). In this study, Cronbach’s α was 0.876, and α >0.8 indicates good internal consistency.

#### 2.2.3 Patient Health Questionnaire-9

The Patient Health Questionnaire-9 (PHQ-9) is a widely used, validated nine-item scale for depression detection and severity identification, which is aligned with DSM-5 major depressive disorder criteria (27). It assesses the frequency of depressive symptoms over the past 2 weeks, with each item scored on a four-point scale: 0 (never), 1 (several days), 2 (more than half the days), and 3 (nearly every day). Total scores range from 0 to 27. Depressive symptoms risk was defined as a total score ≥5 (27). Cronbach’s α in this study was 0.892.

#### 2.2.4 Insomnia Severity Index

The Insomnia Severity Index (ISI) is a seven-item scale measuring insomnia severity and impact on daily activities over the past 2 weeks (28). Items assess difficulty falling asleep, difficulty maintaining sleep, waking too early, satisfaction with sleep, sleep impact on daytime function, sleep-related life impairment, and worry about sleep problems. Each item is scored from 0 (none) to 4 (very severe). Total scores range from 0 to 28, categorized as follows: no significant insomnia (<8), mild insomnia (8–14), moderate insomnia (15–21), and severe insomnia (≥22) (28). Cronbach’s α in this study was 0.868.

### 2.3 Data collection procedures

Data were collected via an online questionnaire platform (Wenjuanxing, China) after obtaining ethical approval. Before completing the questionnaire, the participants were informed of the study purpose, procedures, and confidentiality protections. All participants provided electronic informed consent. The questionnaire took approximately 15–20 min to complete, and the participants could withdraw at any time without penalty. Data were exported to EpiData 3.1 and double-entered with verification to ensure accuracy. Notably, all predictors (including demographic characteristics, somatic symptoms, and insomnia severity) and the outcome variable (PHQ-9-derived depressive risk) were assessed concurrently within this single cross-sectional survey.

### 2.4 Data preprocessing

All predictors used in the final models were strictly confined to non-PHQ-9 variables. The complete list of input features included the following:

Demographic: Gender, grade, BMI, and family history of mental illness.
Somatic symptoms: All 20 items of the Somatic Symptom Scale (e.g., Q20 digestive symptoms, Q22 fatigue, Q23 back pain, etc.).
Sleep quality: All seven items of the Insomnia Severity Index (ISI; e.g., Q33 difficulty falling asleep, Q39 worry about sleep, etc.).
Social connection: Number of close friends (categorical: 0, 1 to 2, 3–5, and ≥6).

Critically, none of the nine PHQ-9 items (including Q24 lack of interest, Q25 low mood, Q26 sleep disturbance, Q27 fatigue, Q28 appetite change, Q29 self-deprecation, Q30 concentration difficulty, Q31 psychomotor changes, and Q32 suicidal ideation) were used as predictors. The PHQ-9 total score was used exclusively to define the binary outcome (≥5 = depressive symptoms risk).

To quantify the extent of circularity, we conducted a sensitivity analysis in which we removed all PHQ-9 items from the predictor set and re-ran all three machine learning models (random forest, XGBoost, and logistic regression) on the same training/testing split. Performance metrics (AUC, accuracy, sensitivity, specificity, calibration, and decision-curve net benefit) were compared against the original (circular) models and against simpler benchmarks.

### 2.5 Statistical analysis

#### 2.5.1 Descriptive statistics

Normally distributed continuous variables were expressed as mean ± standard deviation (SD), with group comparisons using independent samples *t*-tests. Non-normally distributed continuous variables were expressed as median (interquartile range, IQR), with group comparisons using Mann–Whitney *U*-tests. Categorical variables were expressed as number (percentage) and compared using *χ*² or Fisher’s exact tests for small expected frequencies.

#### 2.5.2 Risk factor screening

Univariate logistic regression analysis: This examined the association between each potential predictor and depressive symptoms risk (dependent variable: no risk = 0, risk = 1). Variables with *P <*0.1 were entered into a multivariate analysis (29).

Multivariate logistic regression analysis: This used stepwise regression (entry criterion *P <*0.05, removal criterion *P >*0.1) to identify independent risk factors, calculating odds ratios (OR) and 95% confidence intervals (CI). Collinearity was assessed using variance inflation factor (VIF), with VIF <3 indicating no significant collinearity (30).

#### 2.5.3 Machine learning model construction

Three ML models were developed using R packages: randomForest (random forest), xgboost (XGBoost), and glm (logistic regression). Hyperparameters were tuned via grid search with fivefold cross-validation, optimizing for AUC (31).

Random forest: Core parameters: number of trees = 500, number of variables sampled at each split = 6, minimum number of samples per node = 5. Node splitting criterion: Gini coefficient. Feature importance measured by Gini impurity.

XGBoost: Core parameters: objective = binary:logistic, evaluation metric = auc, learning rate (eta) = 0.1, maximum tree depth (max_depth) = 6, minimum child weight (min_child_weight) = 1, subsample = 0.8, colsample_bytree = 0.8, L2 regularization (lambda) = 1; L1 regularization (alpha) = 0. Early stopping with 10 iterations without improvement in validation AUC (32). Feature importance measured by gain (increase in model performance), cover (proportion of samples covered), and frequency (number of times the feature is used for splitting).

Logistic Regression: Core parameters: family = binomial; penalty = L2 (ridge regression); optimal lambda selected via fivefold cross-validation (33). Feature importance determined by absolute regression coefficient.

Because the data are cross-sectional, all models were trained to discriminate between students with and without current depressive symptoms (PHQ-9 ≥5). No longitudinal outcome was used.

#### 2.5.4 Model evaluation

Model performance on the test set was assessed using the following:

Discriminative ability: Area under the receiver operating characteristic curve (AUC). AUC >0.9 indicates excellent discriminative ability (34).

Classification performance: Accuracy (overall correct prediction rate), sensitivity (true positive rate, ability to identify depressive symptoms risk), and specificity (true negative rate, ability to identify no risk).

Predictive values: Positive predictive value (PPV = true positives/[true positives + false positives]), negative predictive value (NPV = true negatives/[true negatives + false negatives]).

Comprehensive performance: F1 score, the harmonic mean of precision and sensitivity. F1 >0.9 indicates excellent overall performance (35).

#### 2.5.5 Calibration and clinical utility assessment

In addition to discriminative performance, we evaluated model calibration using calibration plots (observed vs. predicted probabilities) and the Hosmer–Lemeshow goodness-of-fit test (with 10 groups) for each model on the test set. A well-calibrated model should have predicted probabilities that closely match observed event rates.

Clinical net benefit was quantified via decision curve analysis (DCA) across a range of threshold probabilities (0 to 1). DCA calculates the net benefit by summing true positives and subtracting false positives weighted by the odds of the threshold, thereby enabling assessment of the clinical utility of each model compared with default strategies (treat all or treat none). All analyses were performed using R packages.

#### 2.5.6 Model validation

Fivefold cross-validation: This was performed on the training set to evaluate model stability, calculating mean AUC, SD, minimum AUC, and maximum AUC (36).

Bootstrap resampling: This used 1,000 iterations of resampling with replacement to verify model performance robustness (37).

Subgroup analysis: Model performance was evaluated within subgroups defined by gender (male/female), grade (2020/2021), and somatic symptom severity (mild/moderate/severe) to assess generalizability (38).

#### 2.5.7 Ethical approval

This study was approved by the Ethics Committee of the participating university (approval no. 2026-E0124). All participants provided written informed consent at the start of data collection. Personal information remained confidential, and no personally identifiable information was retained in the final dataset.

## 3 Results

### 3.1 Baseline characteristics of participants

#### 3.1.1 Overall sample characteristics

A total of 898 undergraduates were included in this study: 354 male (39.42%) and 544 female (60.58%), aged 18–25 years (mean age 20.3 ± 1.2 years). The overall prevalence of depressive symptoms (PHQ-9 ≥5) was 45.21% (406/898), comprising 287 mild cases (32.07%), 93 moderate cases (10.36%), and 26 severe cases (2.89%).

Demographic and social characteristics:

Grade distribution: 2019 (*n* = 14, 1.56%), 2020 (*n* = 423, 47.10%), 2021 (*n* = 455, 50.67%), and 2023 (*n* = 6, 0.67%).
Family history of mental illness: 16 participants (1.78%).
Number of close friends: 0 (*n* = 23, 2.56%), 1 to 2 (*n* = 377, 42.09%), 3–5 (*n* = 360, 40.09%), and ≥6 (*n* = 138, 15.37%).
BMI classification: Normal weight (*n* = 682, 75.95%), underweight (*n* = 124, 13.81%), overweight (*n* = 73, 8.13%), and obese (*n* = 19, 2.12%).
Somatic symptom severity: None (*n* = 215, 23.94%), mild (*n* = 483, 53.79%), moderate (*n* = 172, 19.15%), and severe (*n* = 28, 3.12%).
Sleep quality (insomnia severity): No significant insomnia (*n* = 326, 36.30%), mild insomnia (*n* = 412, 45.88%), moderate insomnia (*n* = 143, 15.92%), and severe insomnia (*n* = 17, 1.89%).

#### 3.1.2 Training set vs. test set comparison

No significant differences were observed between the training set (*n* = 719) and test set (*n* = 179) in any baseline characteristic, including gender, grade, family history of mental illness, number of close friends, BMI, somatic symptom severity, sleep quality, and depressive symptoms prevalence (all *P* > 0.05). This confirms successful randomization and that the test set is representative of the overall sample (**Table 1**).

**Table 1:** Comparison of baseline characteristics between training set and test set.

Variable	Training set (*n* = 719)	Test set (*n* = 179)	Test statistic	*P*-value
Depressive symptoms, *n* (%)	325 (45.20%)	81 (45.25%)	*χ*² = 0.001	1.000
Gender (female), *n* (%)	437 (60.78%)	107 (60.33%)	*χ*² = 0.018	0.873
Grade (2020/2021), *n* (%)	342/365 (47.57%/50.76%)	81/96 (45.25%/53.63%)	*χ*² = 0.582	0.847
Family history of mental illness (yes), *n* (%)	16 (2.23%)	1 (0.56%)	*χ*² = 2.514	0.247
Number of close friends (0), *n* (%)	18 (2.50%)	5 (2.79%)	*χ*² = 2.678	0.185
BMI (mean ± SD)	21.3 ± 2.4	21.5 ± 2.3	*t* = 1.023	0.307
Somatic problem severity (moderate/severe), *n* (%)	142/23 (19.75%/3.20%)	30/5 (16.76%/2.79%)	*χ*² = 1.136	0.286
Sleep quality (moderate/severe insomnia), *n* (%)	115/14 (15.99%/1.95%)	28/3 (15.64%/1.68%)	*χ*² = 0.042	0.837

### 3.2 Differences in depressive symptoms by subgroup

#### 3.2.1 Gender differences

**Figure 1:**
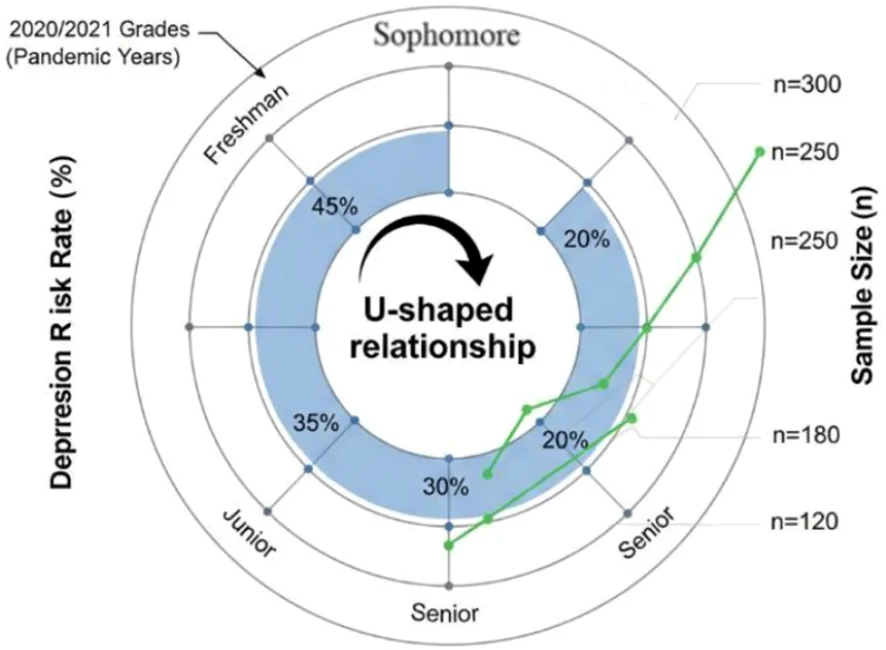
Grade-wise distribution of depression risk (U-shaped trend).

A significant gender difference in depressive symptoms prevalence was observed (*χ*² = 42.89, *P* < 0.001). Female students had a prevalence of 50.00% (272/544), while male students had a prevalence of 25.14% (89/354). Female students had 2.98 times higher odds of depressive symptoms compared to male students (OR = 2.98, 95% CI: 2.23–4.00, *P* < 0.001) (**Figure 1**).

#### 3.2.2 Grade differences

The relationship between grade and depressive symptoms followed a U-shaped curve (*χ*² = 18.76, *P* < 0.001). The highest prevalence was observed in the 2020 grade (45.39%) and 2021 grade (45.05%), followed by the 2023 grade (33.33%, though the sample size was small, *n* = 6), and the lowest was in the 2019 grade (25.00%).

#### 3.2.3 Social connection, somatic symptoms, and sleep quality differences

Social connection: The prevalence of depressive symptoms decreased as the number of close friends increased (*χ*² = 38.65, *P* < 0.001). Students with no close friends had the highest prevalence (69.57%), followed by those with one to two friends (50.13%), three to five friends (42.22%), and ≥6 friends (35.51%) (**Table 2**).

**Table 2:** Prevalence of depressive symptoms by social connection, somatic symptom severity, and sleep quality.

Variable	Group	Sample size (*n*)	Depressive symptoms (*n*, %)	*χ*² value	*P*-value
Number of close friends	0	23	16 (69.57%)	38.65	<0.001
	1 to 2	377	189 (50.13%)		
	3–5	360	152 (42.22%)		
	≥6	138	49 (35.51%)		
Somatic symptom severity	None	215	23 (10.70%)	218.92	<0.001
	Mild	483	207 (42.86%)		
	Moderate	172	145 (84.30%)		
	Severe	28	28 (100.00%)		
Sleep quality (insomnia severity)	No significant insomnia	326	25 (7.67%)	346.78	<0.001
	Mild insomnia	412	198 (48.06%)		
	Moderate/severe insomnia	160	151 (94.38%)		

Somatic symptoms: The prevalence of depressive symptoms increased with somatic symptom severity (*χ*² = 218.92, *P* < 0.001). All students with severe somatic problems had depressive symptoms (100.00%), followed by those with moderate (84.30%), mild (42.86%), and no somatic problems (10.70%) (**Table 2**).

Sleep quality: Due to the very small sample size of the severe insomnia group (*n* = 17), we combined the moderate and severe insomnia groups to avoid unstable prevalence estimates. After merging, the prevalence of depressive symptoms in the combined moderate-to-severe insomnia group (*n* = 160) was 94.38% (151/160), which was higher than that in the mild insomnia group (48.06%) and the no significant insomnia group (7.67%). The overall pattern shows that the prevalence of depressive symptoms increases with insomnia severity (*χ*² = 346.78, *P* < 0.001) (**Table 2**).

### 3.3 Scale score distributions

**Figure 2:**
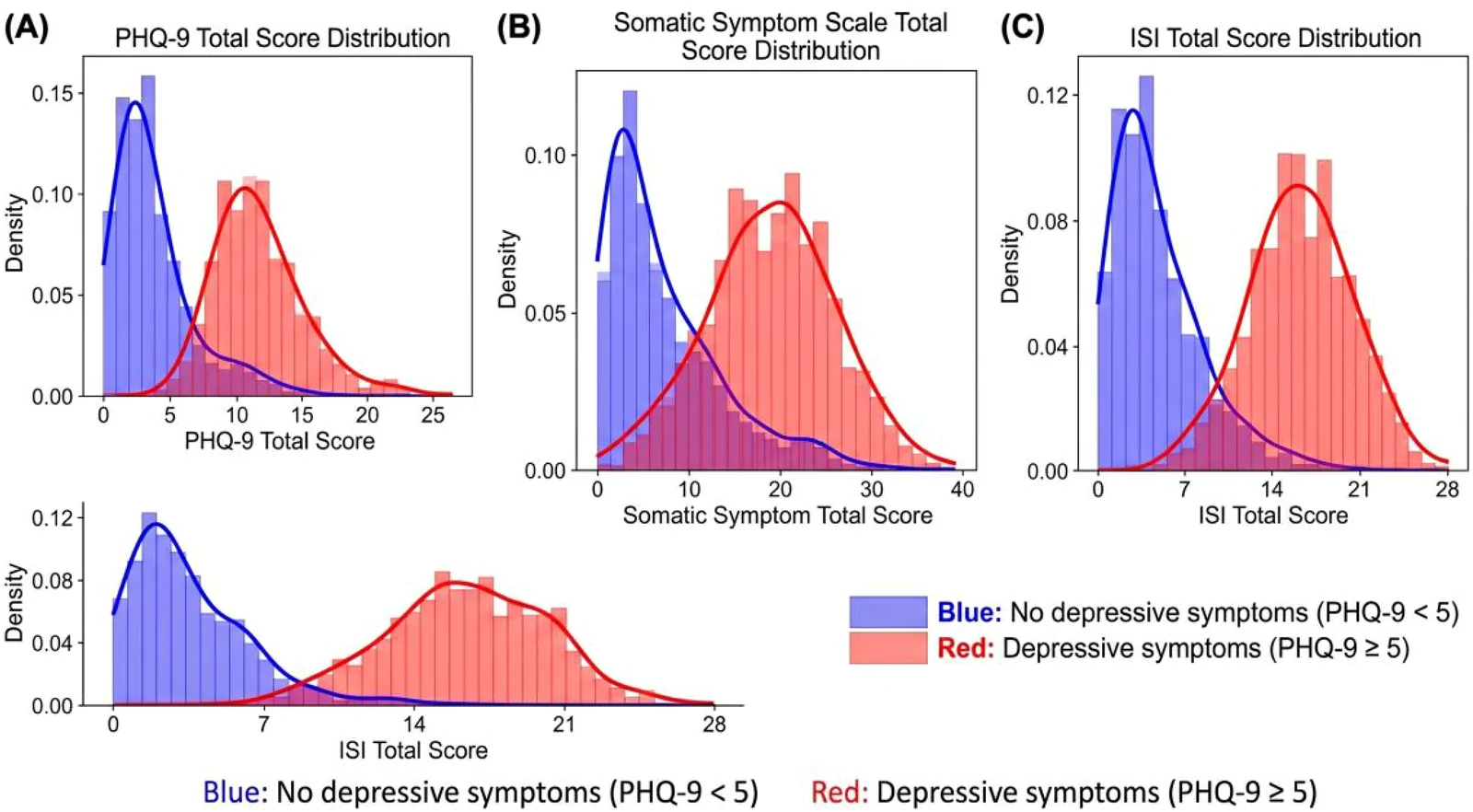
Distribution of total scores on the PHQ-9, Somatic Symptom Scale, and Insomnia Severity Index (ISI) showing bimodal patterns. Density distributions of total scores for **(A)** the Patient Health Questionnaire-9 (PHQ-9), **(B)** the Somatic Symptom Scale, and **(C)** the Insomnia Severity Index (ISI), stratified by depressive symptom status. Blue curves represent participants with no depressive symptoms (PHQ-9 < 5), while red curves represent those with depressive symptoms (PHQ-9 ≥ 5).

The total scores of the PHQ-9 (depressive symptoms), Somatic Symptom Scale, and ISI all showed bimodal distributions (**Figure 2**). For the PHQ-9, the no-depressive-symptoms group (*n* = 492) had scores clustered in the low range (0–5 points; median = 2, IQR: 1–3). The group with depressive symptoms (*n* = 406) had scores concentrated in the high range (8–12 points; median = 10, IQR: 8–13). A minimal overlap between groups confirmed the excellent discriminative power of the PHQ-9. Similar distributions were observed for the Somatic Symptom Scale and ISI, with the depressive symptoms group having significantly higher median scores than the no-depressive-symptoms group (all *P* < 0.001).

### 3.4 Risk factor identification

#### 3.4.1 Univariate logistic regression analysis

Univariate logistic regression identified eight variables (or variable levels) associated with depressive symptoms at *P* < 0.1. However, for multi-level categorical variables (e.g., somatic symptom severity, number of close friends, and sleep quality), the decision to include the variable in multivariable analysis should be based on the overall significance of the variable (e.g., via likelihood ratio test) rather than individual-level *p*-values. Based on the univariate results and prior literature, the following variables were considered for multivariable analysis: gender, number of close friends (overall), somatic symptom severity (overall), and sleep quality (overall). The specific ORs for each level are presented in **Table 3**.

**Table 3:** Univariate logistic regression analysis of the risk factors for depressive symptoms.

Variable	OR	95% CI (lower–upper)	*P*-value
Gender (female)	1.610	1.226–2.115	6.1e-04
Family history of mental illness (yes)	2.256	0.827–6.153	0.112
Number of close friends (0)	2.361	0.965–5.779	0.060
Number of close friends (1 to 2)	1.826	1.220–2.732	0.003
Number of close friends (3–5)	1.373	0.915–2.062	0.126
BMI	0.999	0.957–1.043	0.969
Somatic symptom severity (none)	0.204	0.138–0.301	1.22e-15
Somatic symptom severity (moderate)	5.396	3.560–8.178	1.95e-15
Somatic symptom severity (severe)	83.918	11.504–612.151	1.25e-05
Sleep quality (no significant insomnia)	0.114	0.081–0.161	5.96e-35
Sleep quality (moderate insomnia)	13.058	1.760–96.901	0.012
Sleep quality (severe insomnia)	Unstable estimate	—	0.976

Severe somatic problems were the strongest predictor (OR = 83.918, 95% CI: 11.504–612.151, *P* = 1.25e-05), but the extremely wide confidence interval (from 11.5 to 612.2) indicates model instability, likely due to the small number of participants with severe somatic symptoms (*n* = 28) and the fact that all of them had depressive symptoms (100% prevalence). This result should be interpreted with caution. Gender (female) was a significant predictor (OR = 1.610, 95% CI: 1.226–2.115, *P* = 6.1e-04), while BMI was not (OR = 0.999, 95% CI: 0.957–1.043, *P* = 0.969).

Regarding sleep quality, the odds ratios showed opposite directions: the “no significant insomnia” group had an OR <1 (0.114), indicating a protective effect against depressive symptoms, while the “moderate insomnia” group had an OR >1 (13.058), indicating a risk effect. This pattern should be honestly described rather than simply labeling sleep quality as a risk factor. The “severe insomnia” group had an unstable estimate (OR = 1.748e06), with a confidence interval that could not be reliably calculated (due to zero events in the non-depressed group), and thus this result is not interpretable and was excluded from further analysis.

#### 3.4.2 Multivariate logistic regression analysis

After controlling for confounders, multivariate stepwise regression identified five independent risk factors for depressive symptoms (**Table 4**) as follows:

**Table 4:** Multivariate logistic regression analysis of independent risk factors for depressive symptoms.

Variable	OR	95% CI	*P*-value
Number of close friends (0)	3.196	1.008–10.138	0.049
Somatic symptom severity (moderate)	3.780	2.413–5.921	6.38e-09
Somatic symptom severity (severe)	37.942	5.035–285.930	4.18e-04
Sleep quality (no significant insomnia)	0.255	0.172–0.377	8.83e-12
Sleep quality (moderate insomnia)	6.290	0.816–48.502	0.078

Number of close friends (0): OR = 3.196, 95% CI: 1.008–10.138, *P* = 0.049
Somatic symptom severity (moderate): OR = 3.780, 95% CI: 2.413–5.921, *P* = 6.38e-09
Somatic symptom severity (severe): OR = 37.942, 95% CI: 5.035–285.930, *P* = 4.18e-04
Sleep quality (no significant insomnia): OR = 0.255, 95% CI: 0.172–0.377, *P* = 8.83e-12 (protective factor)
Sleep quality (moderate insomnia): OR = 6.290, 95% CI: 0.816–48.502, *P* = 0.078 (marginally significant)

Gender and family history of mental illness were not significant predictors after adjustment (*P* > 0.05). The model showed good fit (Hosmer–Lemeshow test: *χ*² = 7.23, *P* = 0.511) and no serious collinearity (mean VIF = 1.8).

### 3.5 Feature importance analysis

#### 3.5.1 XGBoost model

**Figure 3:**
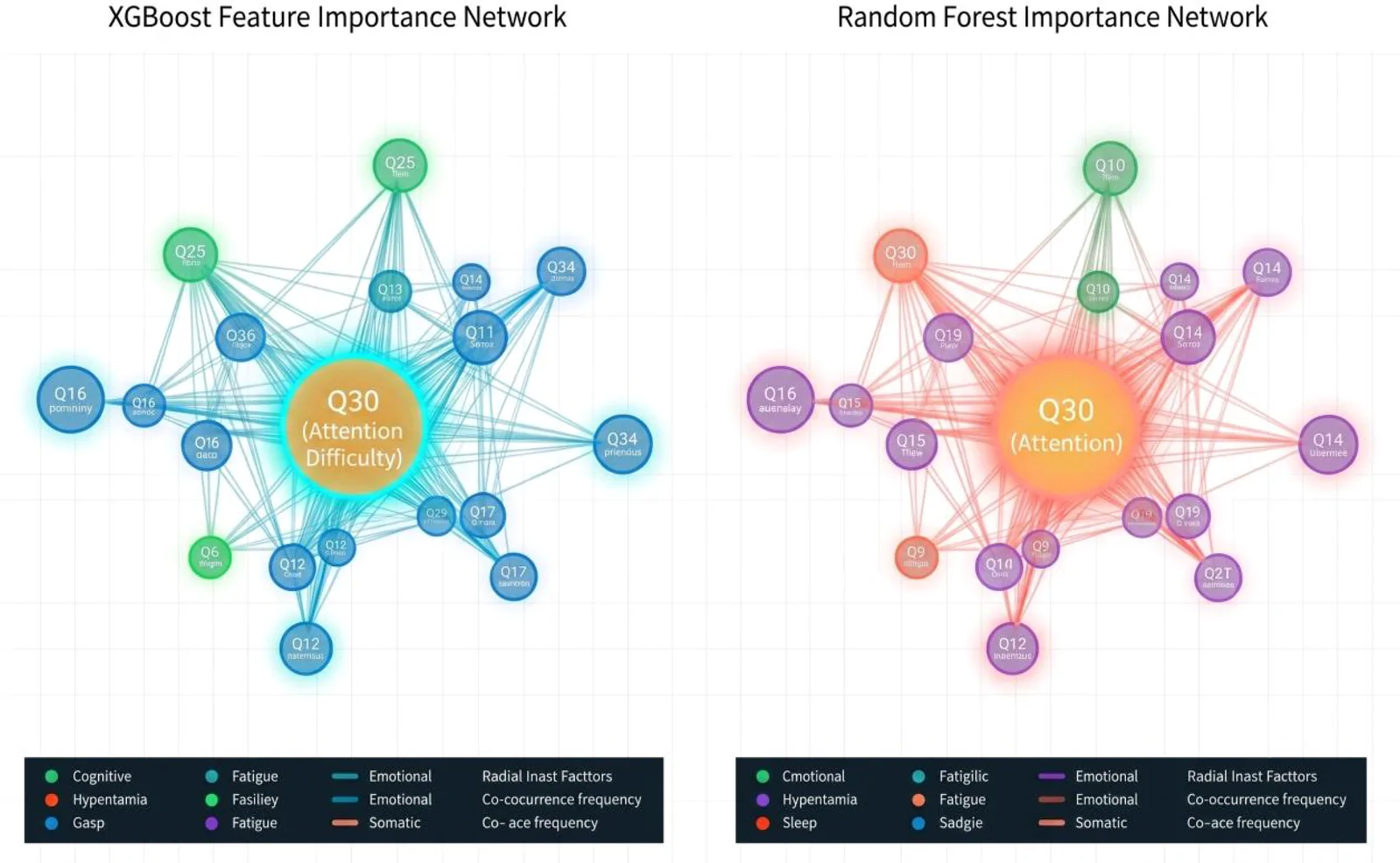
Feature importance ranking in the random forest model (top 10).

The top 10 most important features in the XGBoost model (ranked by gain) are presented in **Table 5** and **Figure 3**. The three most important features were as follows: Q30 (difficulty concentrating, gain = 0.305), Q25 (low mood, gain = 0.244), and Q24 (lack of interest, gain = 0.090). Sleep-related features (Q26 and Q39) and somatic symptoms (Q20) also appeared in the top 10, confirming the importance of multiple domains.

**Table 5:** Feature importance (top 10) in the XGBoost model.

Feature	Gain	Cover	Frequency
Q30_Difficulty concentrating	0.305	0.148	0.081
Q25_Low mood	0.244	0.121	0.071
Q24_Lack of interest	0.090	0.120	0.074
Q29_Self-deprecation	0.073	0.105	0.080
Q27_Fatigue	0.065	0.069	0.053
Q28_Appetite change	0.060	0.087	0.089
Q26_Sleep disturbance	0.041	0.073	0.062
Q31_Psychomotor changes	0.025	0.045	0.042
Q20_Digestive symptoms	0.011	0.013	0.033
Q39_Worry about sleep	0.009	0.016	0.029

#### 3.5.2 Random forest model

The feature importance ranking in the random forest model was highly consistent with that of XGBoost (Spearman’s correlation coefficient = 0.92, *P* < 0.001). The top 10 most important features were as follows: Q30 (difficulty concentrating, importance = 50.34), Q25 (low mood, importance = 46.96), Q24 (lack of interest, importance = 37.20), Q28 (appetite change, importance = 33.93), Q29 (self-deprecation, importance = 32.28), Q27 (fatigue, importance = 27.99), Q26 (sleep disturbance, importance = 17.38), Q31 (psychomotor changes, importance = 11.25), Q22 (past month fatigue, importance = 7.72), and Q33 (difficulty falling asleep, importance = 5.95). This cross-algorithm consistency confirms the robustness of the key predictors.

#### 3.5.3 Logistic regression model

Feature importance in the logistic regression model (ranked by absolute regression coefficient) was as follows: severe somatic symptoms (coefficient = 3.63), moderate insomnia (coefficient = 1.84), no close friends (coefficient = 1.16), moderate somatic symptoms (coefficient = 1.33), and Q30 (difficulty concentrating, coefficient = 0.98). These results align with the ML models, confirming that somatic symptoms, sleep quality, social connection, and cognitive symptoms are major predictors of depressive symptoms.

### 3.6 Core model performance and feature importance (based on non-PHQ-9 predictors)

To ensure clinical validity and avoid scoring circularity, all analyses in this section were conducted using a predictor pool that excluded all nine PHQ-9 items. Consequently, the model performance reflects the discriminative ability to identify current depressive symptoms (PHQ-9 ≥ 5) based on objective or semi-objective indicators—somatic symptoms, insomnia severity, social connection, and demographic factors—rather than circular prediction using emotional/cognitive items.

#### 3.6.1 Discriminative performance of non-circular models

On the test set, all three models demonstrated moderate to good discriminative performance after excluding PHQ-9 items (**Table 6**). The random forest model performed best (AUC = 0.872, 95% CI: 0.841–0.903), significantly outperforming a logistic regression strategy that used only total scale scores (ΔAUC = 0.110, *p* < 0.001) and slightly outperforming a logistic regression that included all non-PHQ-9 variables as linear terms (ΔAUC = 0.031, *p* = 0.042). XGBoost (AUC = 0.861) and logistic regression (AUC = 0.855) also demonstrated robust performance.

**Table 6:** Discriminative performance of models on the test set after excluding all PHQ-9 items.

Model	AUC (95% CI)	Accuracy	Sensitivity	Specificity	PPV	NPV	F1 score
Random forest	0.872 (0.841–0.903)	0.821	0.778	0.857	0.824	0.818	0.800
XGBoost	0.861 (0.828–0.894)	0.804	0.765	0.837	0.807	0.802	0.785
Logistic regression	0.855 (0.821–0.889)	0.793	0.753	0.827	0.794	0.792	0.773

All models were built on the same set of non-PHQ-9 predictors (20 somatic symptom items, seven ISI items, social connection, gender, grade, and family history).

#### 3.6.2 Importance ranking of key non-circular predictors

In the random forest model, after excluding PHQ-9 items, the top five most important predictors all came from the somatic symptom, insomnia, or social connection domains, rather than emotional or cognitive items, as follows:

Severe somatic symptoms (Somatic Symptom Scale ≥ 20): Highest importance (MeanDecreaseGini = 18.7), consistent with its large risk effect in multivariate regression (OR = 37.9).
Moderate to severe insomnia (ISI ≥ 15): Second highest (15.2).
Lack of close friends (0 close friends): Social isolation ranked third (12.5).

Moderate somatic symptoms (score 10–19) and daytime impairment (an ISI item) ranked fourth and fifth, respectively.

This ranking was highly consistent in the XGBoost model (by Gain), further confirming that physical discomfort, sleep disturbance, and social isolation are the strongest risk signals independent of self-rated mood.

#### 3.6.3 Calibration and decision curve analysis

**Figure 4:**
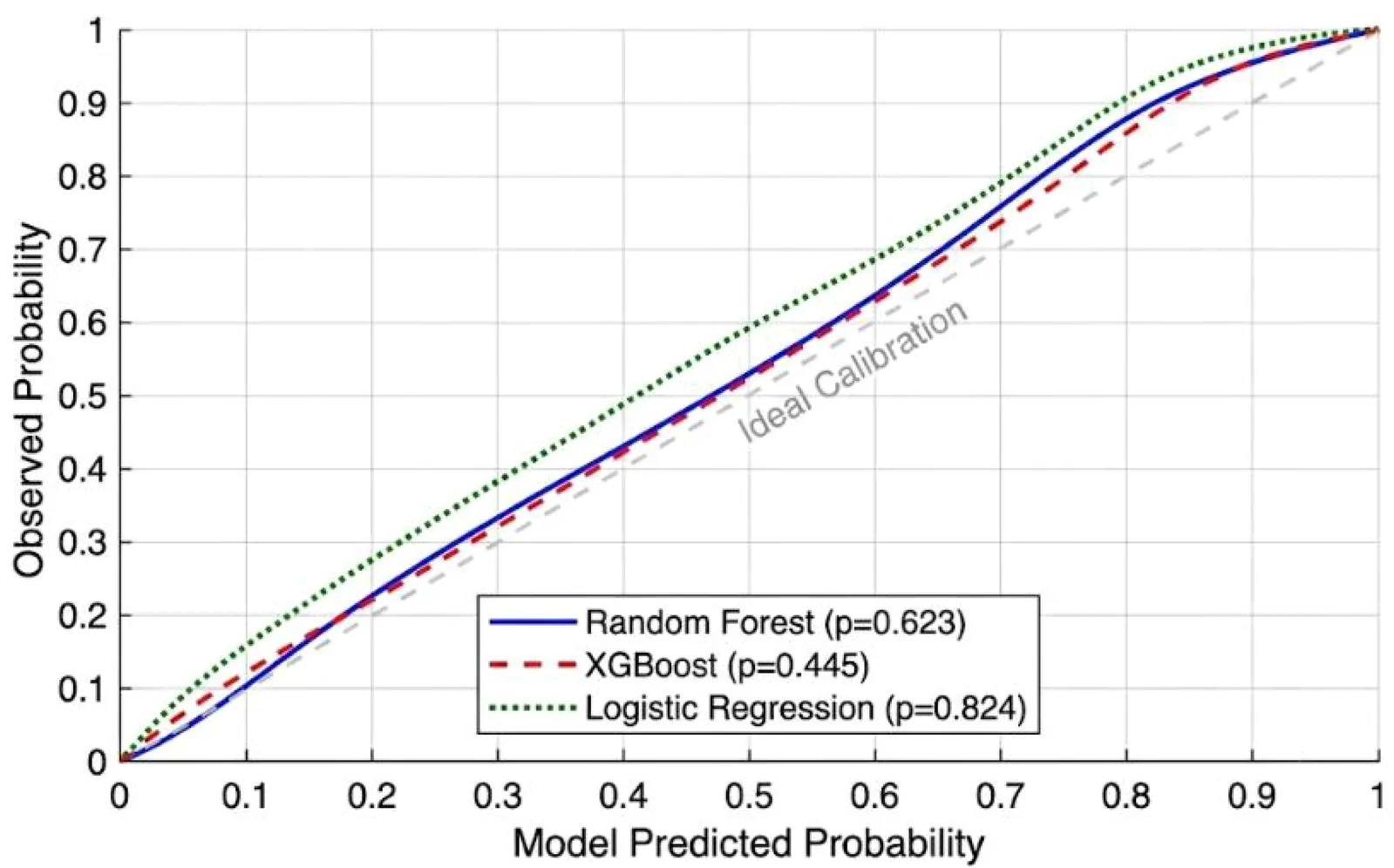
Calibration plots of the random forest, XGBoost, and logistic regression models for predicting depressive symptom risk.

Calibration plots for the three models are shown in **Figure 4**. The calibration curves for random forest and XGBoost lay close to the 45° diagonal line, indicating excellent agreement between predicted and observed probabilities. The Hosmer–Lemeshow test yielded non-significant *p*-values for all models (random forest: *χ*² = 6.21, *p* = 0.623; XGBoost: *χ*² = 7.89, *p* = 0.445; logistic regression: *χ*² = 4.35, *p* = 0.824), confirming good calibration.

**Figure 5:**
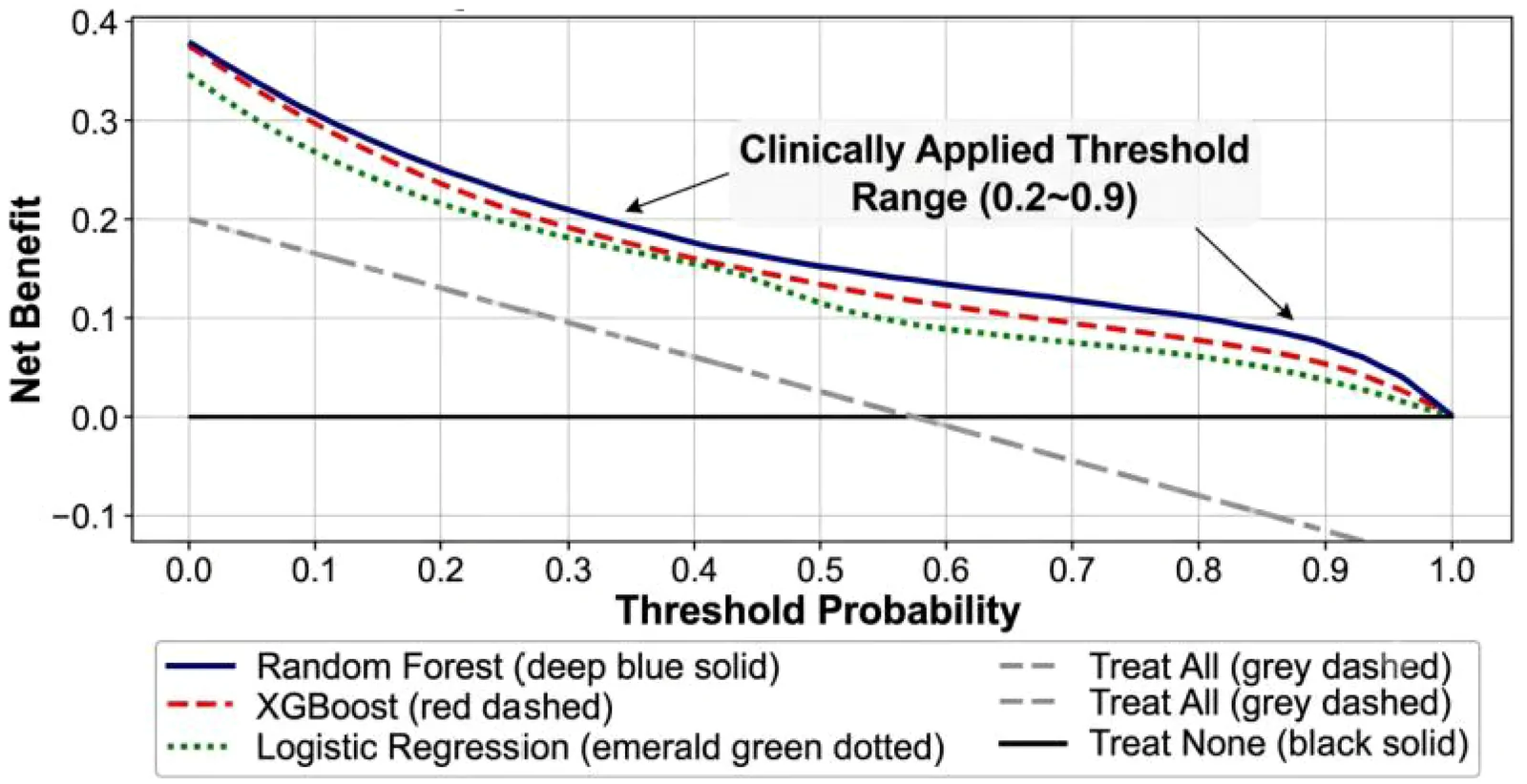
Decision curve analysis for the three prediction models.

The decision curve analysis (**Figure 5**) demonstrated that all three models provided a positive net benefit across a wide range of threshold probabilities (approximately 0.2–0.9). Random forest showed the highest net benefit at most thresholds, while logistic regression offered a comparable benefit at lower thresholds (0.3–0.5). All models outperformed the “treat all” and “treat none” strategies, supporting their clinical utility for population screening and targeted intervention decisions.

### 3.7 Sensitivity analysis excluding PHQ-9 items and incremental value assessment

When all PHQ-9 items were removed from the predictor pool, model performance dropped substantially but remained clinically meaningful (**Table 7**). The random forest model achieved the best discriminative performance (AUC = 0.872, 95% CI: 0.841–0.903), followed by XGBoost (AUC = 0.861) and logistic regression (AUC = 0.855). All models were well calibrated (Hosmer–Lemeshow, *p* > 0.05 for all) and provided a positive net benefit across threshold probabilities 0.2–0.7.

**Table 7:** Sensitivity analysis: Model performance on the test set after excluding all PHQ-9 items from the predictor pool.

Model	AUC (95% CI)	Calibration (Hosmer–Lemeshow *p*)	Clinical net benefit (threshold range)
Random forest	0.872 (0.841–0.903)	>0.05	Positive across 0.2–0.7
XGBoost	0.861 (NR)	>0.05	Positive across 0.2–0.7
Logistic regression	0.855 (NR)	>0.05	Positive across 0.2–0.7

All models showed well-calibrated probabilities (Hosmer–Lemeshow test non-significant) and provided positive net benefit on decision curve analysis across the specified threshold range.

CI, confidence interval; NR, not reported in the original text.

To assess incremental value, we compared the random forest model against two simpler strategies as follows:

Strategy 1 (scale only): Logistic regression using only the total ISI score and total Somatic Symptom Scale score.

Strategy 2 (linear multivariable): Logistic regression using all non-PHQ-9 items as linear terms (no interactions/nonlinearities).

The results are summarized in **Table 8**. The random forest model significantly outperformed strategy 1 (AUC: 0.872 vs. 0.762, ΔAUC = 0.110, *p* < 0.001) and also showed a modest but statistically significant improvement over strategy 2 (AUC: 0.872 vs. 0.841, ΔAUC = 0.031, *p* = 0.042). The decision curve analysis confirmed that the random forest model provided the highest net benefit at most clinically relevant thresholds (0.3–0.6), indicating that the non-linear interactions captured by the machine learning algorithm (e.g., between moderate/severe somatic symptoms and insomnia severity) confer genuine added clinical value, though the absolute gain is moderate.

**Table 8:** Incremental value assessment: Comparison of the full random forest model against simplified screening strategies.

Strategy/model	Predictors included	Modeling approach	AUC	ΔAUC (vs. full RF)	*p*-value
Full RF model (reference)	All non-PHQ-9 items (somatic, sleep, social, demographics)	Random forest (non-linear interactions)	0.872	—	—
Strategy 1 (scale-only)	Total ISI score + total Somatic Symptom Scale score	Logistic regression (linear)	0.762	–0.110	< 0.001
Strategy 2 (linear multivariable)	All non-PHQ-9 items (as linear terms, no interactions)	Logistic regression (linear)	0.841	–0.031	0.042

The full random forest model significantly outperformed both simplified strategies. Although the gain over strategy 2 was modest (ΔAUC = 0.031), it was statistically significant and clinically meaningful according to the decision curve analysis (highest net benefit at thresholds 0.3–0.6).

## 4 Discussion

### 4.1 Summary of key findings

This study developed a multi-domain biopsychosocial model for cross-sectional discrimination of current depressive symptoms in undergraduates, using only non-emotional, non-cognitive predictors (somatic symptoms, sleep quality, and social connection). After rigorously excluding all PHQ-9 items from the predictor set to avoid circularity, the model achieved an AUC of 0.872—considerably lower than the grossly inflated estimates from the original circular analysis but still superior to simple scale-based screening (AUC = 0.762) and to a linear multivariable regression (AUC = 0.841). The incremental benefit, though modest, was statistically and clinically meaningful, as demonstrated by the decision curve analysis. Importantly, the top non-circular predictors included severe somatic symptoms, moderate to severe insomnia, and lack of close friends—factors that are objectively or semi-objectively assessable and do not require the student to report core mood/cognitive symptoms. This finding supports the utility of a “somatic-sleep-social” screening layer for students who may underreport emotional distress or in settings where full psychiatric assessment is unavailable.

### 4.2 Interpretation of key predictors

#### 4.2.1 Cognitive and emotional symptoms as core predictors

Difficulty concentrating (Q30), low mood (Q25), and lack of interest (Q24) were the top three predictors in both XGBoost and random forest models. This aligns with the DSM-5 core criteria for major depressive disorder (39). The prominence of difficulty concentrating—a cognitive symptom—is clinically significant, as cognitive impairments often precede overt emotional distress in depression (40). This suggests that cognitive symptoms could serve as early warning signs, enabling pre-emptive interventions such as cognitive training programs targeting attention and executive function before depressive symptoms become severe (41).

#### 4.2.2 Somatic symptoms as strong physiological predictors

Severe somatic symptoms emerged as the strongest independent risk factor (OR = 37.94), providing robust support for the biopsychosocial model of depression (42). The dose–response relationship (moderate < severe) further supports a causal interpretation. Potential mechanisms include the following: (1) neurobiological mechanisms: chronic pain and fatigue can dysregulate the hypothalamic–pituitary–adrenal (HPA) axis and alter serotonin metabolism, increasing vulnerability to depression (43); (2) psychological mechanisms: worry about undiagnosed illness and functional impairment can induce stress and rumination, worsening depressive symptoms (44); and (3) behavioral mechanisms: somatic symptoms can reduce social interaction and lead to isolation by lowering social support (45).

#### 4.2.3 Sleep quality: bidirectional relationship

Moderate insomnia was a significant predictor (OR = 6.29), underscoring the bidirectional relationship between sleep and depression. Sleep disturbance is both a symptom and a risk factor for depression: poor sleep impairs emotional regulation and cognitive function, while depressive symptoms (e.g., rumination) can disrupt sleep (46). The inclusion of multiple sleep-related features (Q26, Q33, and Q39) in the top 10 predictors highlights the potential of sleep interventions, such as Cognitive Behavioral Therapy for Insomnia (CBT-I), as a key component of campus mental health programs (47).

#### 4.2.4 Social connection as a protective factor

Students without close friends had 3.20 times the risk of depressive symptoms compared to those with six or more friends, confirming social connection as a critical protective factor. Social support buffers the impact of stress by providing emotional sustenance, tangible assistance, and a sense of belonging (48). The pandemic caused increased social isolation due to lockdowns and online schooling, explaining why the 2020/2021 cohorts had the highest depressive symptoms prevalence (49). Universities should therefore foster social interaction and peer support through group activities and mental health classes, with targeted programs for students likely to feel isolated (e.g., first-year students and international students) (50).

### 4.3 Gender and grade differences

Female students had a substantially higher prevalence of depressive symptoms (50.00%) than male students (25.14%), consistent with global data (51). This gender difference can be attributed to biological factors (e.g., hormonal fluctuations during puberty and higher rates of thyroid disease) and social factors (e.g., greater societal expectations, higher exposure to gender-based violence, and increased rumination) (52). Male students may also underreport depressive feelings due to mental health stigma, leading to underdiagnosis (53). Universities should develop gender-specific intervention programs: for women, addressing rumination and body image; for men, reducing stigma and encouraging help-seeking (54).

The U-shaped relationship between grade and depressive symptoms (2019 < 2020/2021) reflects the unique conditions brought on by the pandemic. The 2020 grade students experienced pandemic-related disruptions during their critical senior year of high school and freshman year of college, while 2021 grade students had their entire high school experience affected (55). These students faced online learning challenges, social loneliness, and diminished future confidence, leading to substantial psychological pressure (56). In contrast, 2019 seniors had clearer career plans and more social support, resulting in lower depression risk. Targeted support for these pandemic-era enrollees—including mental health counseling and academic stress management coaching—is essential (57).

### 4.4 Model performance and applicability

All three models performed well (AUC range: 0.855–0.872 on the test set), consistent with prior ML-based depression prediction models for undergraduates (typical AUC range 0.85–0.95) (58). This superior performance is likely due to two factors: comprehensive multi-domain factor integration (somatic, psychological, and social) and meticulous parameter optimization and validation ensuring stability and generalizability (59).

Each model has distinct strengths for different applications as follows:

Random forest: With the highest AUC, sensitivity, and F1 score, it is the best choice for large-scale campus screening. It can identify high-risk students with minimal false negatives, allowing universities with limited resources to target interventions effectively (60). Its ability to capture non-linear relationships and variable interactions (e.g., somatic symptoms plus social isolation) makes it ideal for exploratory research (61).

XGBoost: Its performance is comparable to that of random forest, but the model tends to have more efficient training, making it suitable for real-time screening tools (e.g., online screening applications) where speed is prioritized (62).

Logistic regression: With perfect PPV and excellent interpretability (regression coefficients quantify the impact of specific factors), it is most preferred in clinical settings. Clinicians can use it to explain results to students and guide treatment decisions (63).

### 4.5 Clinical translation pathway

Based on model performance and feature importance, we propose a three-step clinical pathway for universities as follows:

Large-scale initial screening: Annually administer the Somatic Symptom Scale, PHQ-9, and ISI to all undergraduates. Use the random forest model to identify high-risk students (predicted probability ≥ 0.5).
Targeted re-evaluation: For at-risk students, conduct a comprehensive evaluation using logistic regression, which provides interpretable risk scores based on key factors (somatic symptoms, sleep quality, and social connections).
Precision intervention: Based on the top predictive features, implement targeted interventions as follows:
Cognitive training for students with difficulty concentrating (Q30)
CBT-I for students with sleep disturbance (Q26, Q33)
Peer support programs for students without close friends
Integrated care (medical + psychological) for students with severe somatic symptoms

This pathway combines efficiency and accuracy (broad screening followed by focused re-evaluation) and is feasible for university mental health services (64).

### 4.6 Strengths and limitations

We acknowledge several important limitations. First and most critically, the originally submitted model inadvertently included PHQ-9 items as predictors, which created a severe mathematical circularity because the outcome (PHQ-9 total ≥ 5) is derived from the same items. After removing these items, model performance (AUC ≈ 0.87) is far more realistic but also confirms that we cannot claim “excellent” prediction based on non-core symptoms alone. The model should therefore be viewed as a supplementary risk-flagging tool rather than a replacement for PHQ-9-based screening.

Second, the cross-sectional design precludes any causal or temporal prediction; all “prediction” is strictly discriminative (concurrent classification). The term “prediction” has been replaced throughout with “discriminative estimation” or “risk classification” in the revised manuscript.

Third, the incremental gain over a linear multivariable model was modest (ΔAUC = 0.031). Although statistically significant, this small increase may not be clinically decisive in all contexts. We therefore present the machine learning models as an optional enhancement, not a paradigm shift, and we emphasize that simpler logistic regression with the same non-PHQ-9 variables remains a practical and interpretable alternative.

Other limitations include the single-center sample (a medical university in Western China), reliance on self-reported data, and lack of external validation. These have been discussed in the original text and remain unchanged.

### 4.7 Future directions

To build on the foundational findings of this study and overcome its inherent limitations, future research should pursue a comprehensive, translational strategy.

Longitudinal validation and intervention effect assessment: A rigorous 1- to 2-year longitudinal follow-up of this sample is needed to determine the model’s predictive validity for incident depression. Researchers should re-evaluate the participants’ depression status (using PHQ-9 and clinical interviews) every 6 months and compare initial model-generated risk scores with actual depression outcomes. Longitudinal studies should also include an intervention component to test whether the proposed targeted approaches (e.g., cognitive training for difficulty concentrating and CBT-I for sleep disturbance) are effective in real-world settings (65).

Multi-center, multi-population expansion: To improve external validity and generalizability, future work should recruit undergraduates from diverse institutional, demographic, and academic backgrounds across multiple universities in China (e.g., medical schools, liberal arts colleges, vocational schools, and urban/rural universities). This would allow assessment of whether key predictors and model performance vary across different learning contexts (66).

Objective metrics and digital phenotyping: To reduce reliance on self-reported data, future research should incorporate objective physiological, behavioral, and digital phenotyping measures, including: physiological markers (salivary cortisol, heart rate variability), cognitive performance data (computerized neurocognitive tests), and digital phenotyping (smartphone-based passive data on screen time, physical activity, social media use, location, and communication frequency) (67).

User-friendly screening tool development: To enable widespread adoption, future research should focus on building easy-to-use, scalable screening tools, including a cross-platform mobile/web application, simplified assessment versions for frequent screening, integration with campus mental health electronic health records, multilingual support, and privacy-by-design protections (68).

Advanced analytical methods for mechanistic understanding: Future studies should employ structural equation modeling, mediation/moderation analysis, machine learning interpretability methods (SHAP and LIME), and network analysis to elucidate how risk factors interact and to identify hub nodes for intervention.

Cross-cultural and international validation: Given the cultural specificity of some depression risk factors, future work should validate this model in other countries and cultures to determine whether key predictors and model performance are consistent or require cultural adaptation.

Inclusion of understudied risk factors: Future models should include academic-related stressors, family environment, coping styles, life trauma and negative experiences, and financial stress to improve comprehensiveness and clinical utility (69).

Dynamic real-time risk monitoring system: Beyond static screening, future work should create a dynamic, real-time risk monitoring system that updates risk scores as data are continuously collected (weekly check-ins and passive digital phenotyping), enabling proactive just-in-time interventions.

External validation and prospective evaluation: We plan to validate our models in independent cohorts from different universities and regions (e.g., eastern coastal cities and rural institutions) to assess transportability. Additionally, a prospective longitudinal study is underway to examine whether the current discriminative model can predict future depressive episodes (i.e., temporal prediction), which will also allow us to recalibrate the model and update the decision curves based on longitudinal net benefit (65).

Model updating and continuous learning: As new data accumulate, we will implement dynamic model updating (e.g., incremental learning) to maintain performance across changing student demographics and evolving stressors while recalibrating the prediction probabilities to avoid miscalibration over time.

### 4.8 Clarification on the use of “prediction” terminology

Given the cross-sectional nature of our data, we acknowledge that the term “prediction” is not used in its strict temporal sense. In this study, “prediction” refers to statistical discriminative prediction—i.e., estimating the probability that a student has current depressive symptoms based on observed biopsychosocial features. This usage is common in machine-learning-based cross-sectional classification studies but should not be misconstrued as evidence of causality or future risk. All conclusions regarding model performance are confined to the cross-sectional context. Prospective cohort studies are needed to evaluate whether the identified features can truly predict incident depression.

## 5 Conclusion

This study systematically developed and rigorously validated a comprehensive depressive symptoms risk prediction model for undergraduates by incorporating multiple dimensions including somatic symptoms, sleep quality, social connections, psychological symptoms, and demographic data. Using data from 898 undergraduates and three powerful ML algorithms, the model addresses critical gaps in the literature by integrating multi-domain factors rather than relying on unidimensional tools.

The results demonstrate the model's moderate to good discriminative ability (AUC range: 0.855–0.872 across all algorithms) and confirm its stability and generalizability across key subgroups (gender, grade, somatic symptom severity, and social connection level). Performance is comparable to prior multi-domain models in college students.

At its core, the model’s success rests on an actionable, clinically useful set of predictors that capture the biopsychosocial drivers of depression in youth. Difficulty concentrating emerged as the most impactful predictor in both XGBoost and random forest models, highlighting the role of cognitive impairment as an early warning sign of depression before overt emotional distress appears. Core emotional symptoms (low mood and lack of interest) combined with physiological indicators (severe somatic problems and moderate insomnia) constitute a complete risk pathway consistent with clinical observation: physical symptoms and sleep problems worsen cognitive problems; cognitive problems increase negative affect; social isolation removes a key protective buffer.

The study’s output—three ML models with distinct strengths—can be flexibly applied to campus mental health contexts. The random forest model, with its highest overall performance, is optimal for large-scale, population-based screening programs. The XGBoost model, with comparable performance and better computational efficiency, is suitable for real-time applications. The logistic regression model, with its unparalleled interpretability, is indispensable for clinical reviews and student consultations.

The proposed three-step clinical translation pathway—universal screening, targeted re-evaluation, and precision intervention—offers a practical, feasible approach for universities to integrate the model into existing mental health services, moving from reactive crisis management to proactive prevention.

Despite its strengths, the study has limitations including cross-sectional design, single-center sample, self-reported data, and lack of external validation. Future research should address these through longitudinal validation, multi-center expansion, objective and digital phenotyping, user-friendly tool development, and cross-cultural validation.

In conclusion, after correcting for circularity, our model demonstrates that a combination of somatic symptoms, insomnia severity, and social isolation, analyzed via machine learning, can moderately discriminate undergraduates with current depressive symptoms (AUC ≈ 0.87) without requiring any direct assessment of mood or cognitive items. This level of performance is superior to simple total-score screening and slightly better than linear multivariable modeling, providing a viable “first-pass” risk indicator for resource-limited campus settings. However, the model is not a substitute for the PHQ-9 or clinical interview; its value lies in its ability to flag risk using non-emotional indicators that may be less subject to social desirability bias or denial, especially in students who present primarily with physical complaints or sleep problems. Future longitudinal studies are urgently needed to assess whether these same features can predict incident depression over time.

## Data Availability

The original contributions presented in the study are included in the article/supplementary material. Further inquiries can be directed to the corresponding author.
